# Genome-Wide Identification and Expression Analysis of *Aspartic proteases* in *Populus euphratica* Reveals Candidates Involved in Salt Tolerance

**DOI:** 10.3390/plants14131930

**Published:** 2025-06-23

**Authors:** Peiyang He, Lifan Huang, Hanyang Cai

**Affiliations:** 1 College of Life Sciences, Fujian Agriculture and Forestry University, Fuzhou 350002, China; 15090921733@163.com; 2 Jinshan College, Fujian Agriculture and Forestry University, Fuzhou 350002, China; 13905944586@163.com

**Keywords:** *Populus euphratica*, gene family, *Aspartic proteases*, salinity tolerance

## Abstract

Aspartic proteases (APs) are among the four primary families of proteolytic enzymes found in plants, and they are essential for both stress response mechanisms and developmental activities. While the *AP* gene family has been studied in model plants like *Arabidopsis*, its characterization in woody species-particularly in extremophytes like *Populus euphratica*, remains limited. Moreover, the potential involvement of *APs* in salt tolerance mechanisms in trees is yet to be explored. In this research, 55 *Pe*APs were discovered and categorized into three distinct classes based on their conserved protein structures. The phylogenetic analysis revealed potential functions of *AP* genes derived from *Arabidopsis thaliana*, *V. vinifera*, and *P. euphratica*. Our findings indicate that *PeAP* possesses a well-conserved evolutionary background and contains numerous highly variable regions, making it an excellent candidate for the identification and systematic examination of woody trees. Additionally, motifs frequently found in aspartic proteases within the genome of *P. euphratica* may be linked to functional *PeAP*s. It appears that *PeAPs* are associated with specific gene functions. These genes are influenced by cis-elements, which may play a role in their responsiveness to phytohormone, stress adaptation maybe changed to these genes are regulated by cis-elements that may mediate their responsiveness to phytohormones, abiotic stress, and developmental cues. Our research offers the initial comprehensive analysis of the *AP* family in *P. euphratica*, emphasizing its potential functions in adapting to salt conditions. The findings uncover candidate *PeAPs* for genetic engineering to enhance salinity tolerance in woody crops.

## 1. Introduction

Soil salinity has emerged as one of the most critical abiotic stresses threatening global forest ecosystems, with recent estimates suggesting that over 20% of irrigated agricultural lands worldwide are affected by secondary salinization [1]. This environmental challenge is particularly acute for woody perennials, where prolonged exposure to saline conditions disrupts physiological processes ranging from nutrient acquisition to vascular development [2,3,4]. Among tree species, *Populus euphratica* has garnered significant scientific attention due to its remarkable capacity to thrive in saline-alkali soils with NaCl concentrations exceeding 400 mM—a trait attributed to its sophisticated ion homeostasis mechanisms [5]. Recent studies have elucidated several components of this halophytic adaptation system, including the SOS pathway-mediated Na^+^ exclusion, enhanced antioxidant enzyme activities, and developmental plasticity in root architecture [4]. However, despite these advances, the proteolytic regulation underlying *P. euphratica*’s salt tolerance remains largely unexplored, particularly concerning the aspartic protease (AP) gene family, which has been associated with stress responses across various plants [6,7].

Aspartic proteases represent one of four major classes of proteolytic enzymes in plants, characterized by their conserved biological structure containing two catalytic aspartate residues in the characteristic DTG/DSG motifs [8,9]. Phylogenetic analyses reveal that this gene family has undergone significant expansion during land plant evolution, with modern species displaying remarkable diversity in *AP* gene copy numbers—from 51 members in *Arabidopsis* to 96 in *Oryza sativa* [10,11]. Beyond their canonical roles in protein turnover and processing, plant *APs* have been increasingly recognized as key regulators of stress responses through various mechanisms. In *Arabidopsis thaliana* ectopic expression of a grape aspartic protease gene, *AP13*, improves resistance to powdery mildew by promotes the SA dependent signal transduction pathway, but suppresses the JA signal transduction pathway [12]. In addition, *ASPG1* (NP_188478) the *ASPARTIC PROTEASE IN GUARD CELL 1* gene whose over expression conferred drought avoidance via ABA-dependent signaling in *Arabidopsis* [13]. Perhaps most intriguingly, certain AP isoforms in *Arabidopsis* (*AtPCS1*) and sweet potato (*SpAP1*) have been demonstrated to influence developmental processes ranging from embryogenesis to leaf senescence [14,15], suggesting evolutionary co-option of proteolytic functions for regulatory purposes.

In the context of *P. euphratica*’s salt tolerance, several molecular adaptations have been characterized that may intersect with AP functionality. The species exhibits sophisticated transcriptional reprogramming during salt stress, involving *NAC* and *WRKY* transcription factors that regulate ion transporter genes such as *NHX1* and *HKT1* [16,17]. Post-translational modifications (PTMs) also play crucial roles, with recent studies identifying stress-responsive deubiquitination of some proteins [18] and glycosylation of membrane proteins [19]. Furthermore, while the *Populus trichocarpa* genome project revealed extensive gene family expansion through segmental duplications [20], the evolutionary trajectory and functional specialization of *AP* genes in its extremophyte relative *P. euphratica* have never been systematically investigated.

The current understanding of plant *APs* presents several critical knowledge gaps that this study addresses. First, while *AP* family members have been comprehensively annotated in model herbaceous species like *Arabidopsis* [10] and rice [11], their identification and classification in extremophyte trees like *P. euphratica* remains incomplete, hindering comparative evolutionary analyses across ecological niches. Second, the potential involvement of *APs* in woody plant stress responses has been largely overlooked, despite emerging evidence of their regulatory roles in herbaceous species [21]. Third, the relationship between *AP* gene family expansion through whole genome duplication events [20] and functional diversification in stress adaptation contexts remains unclear. Finally, while post-translational modifications like glycosylation are known to regulate AP activity in some microbials [22], no studies have explored the intriguing possibility that *AP* function might be modulated by glycosylation, which was a key salinity-responsive PTM in *P. euphratica*. These gaps collectively represent a significant limitation in our understanding of plant stress biology, particularly for ecologically and economically important tree species facing increasing salinity pressures [23], where *APs* may serve as underexplored nodes in stress signaling networks.

In this study, we conducted a comprehensive analysis of the complete genome of *P. euphratica*, focusing on the identification and characterization of the *PeAP* gene family. Our investigation delved into the phylogenetic relationships among these genes, as well as their structural properties at the protein level. We also explored transcriptional patterns, the presence of *cis*-acting elements within their promoters, and the subcellular localization of the proteins were encoded by these genes. Additionally, we examined potential interactions that may occur among these proteins. Through this extensive analysis, we identified a specific group of *PeAP* genes that appeared to play significant roles in the ability of medicinal plants to adapt and respond to varying environmental conditions.

## 2. Results

### 2.1. Genome-Wide Identification and Phylogenetic Analysis of P. euphratica AP Gene Family

**Figure 1:**
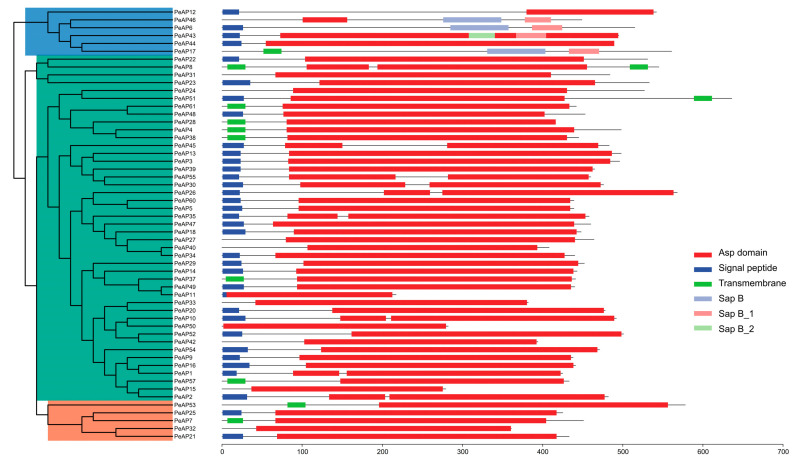
Phylogenetic tree, conserved domain analysis of aspartic proteases gene family in *Populus euphratica*. Individual conserved domains are indicated by different colored boxes.

In order to pinpoint members of the *AP* family within the genus *Populus*, a comprehensive BLASTP analysis was conducted. This analysis utilized previously documented AP protein sequences from *Arabidopsis* as the query for comparison. We discovered and characterized a total of 61 *APs* in *P. euphratica*, using *APs* from *Arabidopsis thaliana* and *Vitis vinifera* as references (Table S1). The findings of this analysis revealed that a significant number of the identified proteins, specifically, a total of 55 *Pe*APs, exhibited high sequence conservation, indicating evolutionary preservation. This conservation aligns with the *APs* that have been previously identified in *A. taliana*. Based on these characteristics, the 55 *Pe*APs, as well as other known AP proteins (Nucellin, CND41 and cardosin A) were categorized into three distinct groups, labeled A, B, and C, similar to those described in *Arabidopsis* and *grape*. Every group had individual conserved domains, which reinforced the relevance of the classification (Figure 1). Approximately 94.5% of the *Pe*AP CDSs encode more than 360 aa long, while *Pe*AP11 (217 aa), *Pe*AP15 (279 aa), and *Pe*AP50 (282 aa) were unique (Table S1). The protein sequences within each category exhibited significant similarities. The *Pe*APs molecular weights ranged between 23.4 and 70.8 kDa. Isoelectric points showed variation from 4.65 to 9.78. Among them, *PeAP51* encodes the heaviest protein at 70.8 kDa, while *PeAP11* encodes the lightest at 23.4 kDa (Table S1). The characteristics of the *PeAPs* closely resemble those of APs found in other plant species [10,24], suggesting that the functions of these *Pe*APs have been evolutionarily preserved.

### 2.2. Phylogenetic Analysis of the PeAP Proteins

**Figure 2:**
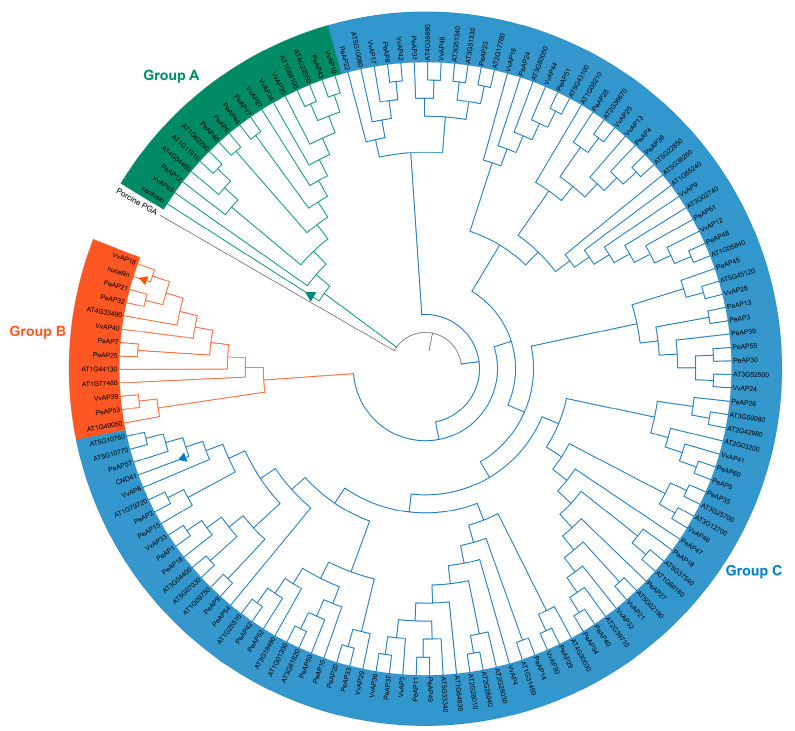
Phylogenetic tree of aspartic proteases in *A. thaliana*, *Vitis vinifera*, and *P. euphratica* constructed by the neighbor-joining method in MEGA-X. The numbers at nodes represent bootstrap values after 1000 iterations. Each group is indicated by a different color.

To investigate the phylogenetic connections among plant *Pe*AP*s*, we created a dataset comprising 51 *A. thaliana*, 30 *V. vinifera*, and 55 *P. euphratica* AP amino acid sequences (Table S2), which were utilized to build a neighbor-joining tree. No assumptions were made regarding ancestral representation, relying solely on the relationships observed among the leaf nodes in unrooted trees. Consequently, the AP*s* were categorized into three distinct groups (A, B, and C), as anticipated in Figure 2. The *Pe*AP proteins demonstrated significant similarity to APs found in module plants. These genes were organized into clades alongside *AtAP*s and *VvAP*s, which exhibited superior bootstrap values. In the A clade, there were 5 *AtAP*s, 5 *VvAPs*, and 6 *PeAP*s, while the B group contained 4, 3, and 5 APs from each respective species. Notably, the C group included 44 *PeAP*s. This information suggests that the *AP* within the *P. euphratica* genome has undergone a distinct biological evolution when compared to those of the module plants. Across all species, the C group was the most populous, comprising 42 *AtAP*s, 22 *VvAP*s, and 44 *PeAP*s. In summary, our findings indicate that the *AP*s present in the *P. euphratica* genome are highly conserved evolutionarily and contain numerous highly variable regions, making them particularly suitable for medical plant identification and systematic research.

### 2.3. Predicted Structure and Conserved Motifs Analysis of PeAP Proteins

To visualize the various protein structures of aspartic proteases, we selected representative proteins with similar amino acid lengths and compared their structures from each group of three plant species for analysis (Figure S1). Groups C proteins have similar structures among diverse plant species. What’s more, these proteins from groups A and B share significant overall structural similarity, certain regions exhibit distinct differences. Furthermore, the Group C protein sequences were highly conserved, although there were minor variations in the folding regions. Considering that the structure and characteristics of proteins influence the traits and roles of organisms, this information implies that the APs from these three plants could share homologous biological functions. The conserved roles of *Pe*APs indicate that they appear to be linked to particular functional guidelines.

**Figure 3:**
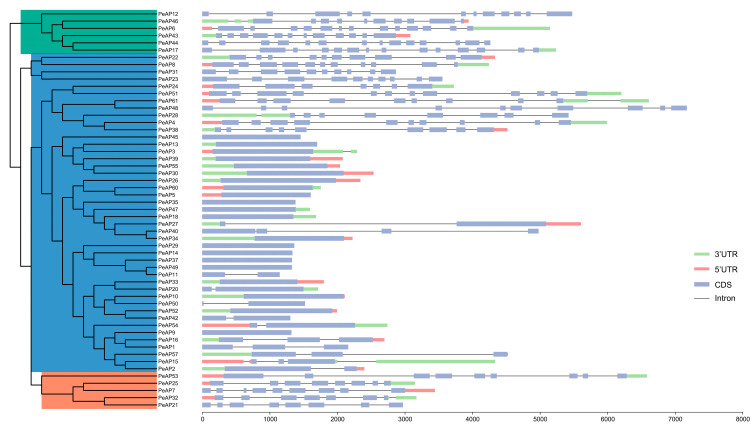
Gene structure of aspartic proteases genes in *P. euphratica*. Untranslated regions (UTR) and coding sequence (CDS) are indicated by green, red and blue frames on the right, respectively. The number on the gray line represents the number of introns. Different colored frames represents different protein motifs, and each motif has its own number.

To explore the evolutionary trajectory of the gene family, a comparison analysis of the gene structures of the *PeAPs* was conducted, as illustrated in Figure 3. Our examination of the genomic DNA sequences showed a variation in the number of introns, ranging from 0 to 13. The *PeAP*s predominantly exhibited highly comparable structures, which were categorized into Groups A and B branches within the NJ tree. All *PeAPs* belonging to Groups A and B displayed similar counts of introns and exons, except for Group C, which included 18 *PeAPs* that had lost introns. Additionally, to uncover potential motifs within the AP family of *P. euphratica*, we utilized the MEME website to predict the protein sequences of all complete *Pe*APs. As a result, we identified 10 distinct motifs from these proteins (Table S3). Apart from the identified motifs, amino acid sequence alignment of part of the sequences of abovementioned *Pe*APs (Figure S2). The group exhibited similar motifs, suggesting that these proteins may share certain common functions. In summary, this analysis reinforced the idea that motifs frequently found in aspartic proteases within the *P. euphratica* genome could be linked to the conserved functions of *Pe*APs, while the *Pe*APs appear to be connected to specific functional roles.

### 2.4. Evolutionary Relationships of AP Genes Between P. euphratica and Arabidopsis

In order to delve deeper into the origins and evolutionary development of *P. euphratica AP* genes, we examined the comparative synteny map that exists between the genomes of *P. euphratica* and *Arabidopsis*. As one of the key model plant species, *Arabidopsis* plays a significant role, especially concerning *AP* genes, as the functions of several of these genes have been thoroughly studied. Thus, through comparative genomics, we can determine the origin and diversification of *P. euphratica AP*s based on their *Arabidopsis* homologs.

**Figure 4:**
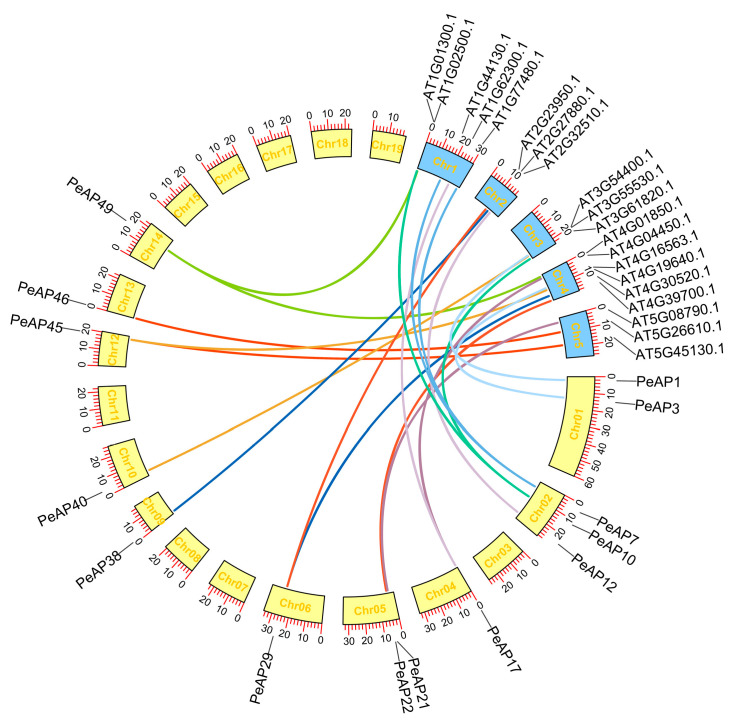
Collinearity analysis of aspartic protease genes between *A. thaliana* and *P. euphratica*. Colored bars denote syntenic regions between *Arabidopsis* and *P. euphratica* AP chromosomes.

Large-scale syntenies containing 14 *AP* genes in populus and 20 in *Arabidopsis* were identified (Figure 4). In addition, 15 genes within the *Arabidopsis* genome, which were not classified as *AP* genes, were discovered to exhibit synteny with genes from grape *P. euphratica* (Figure 4 and Table S1). Concerning the individual correspondences between *Populus* and *Arabidopsis AP* genes, the syntenic relationships were clear-cut and comprised the following orthologous pairs: *PeAP1*-At3g54400, *PeAP10*-At3g61820, *PeAP7*-At1g77480, *PeAP7*-At1g44130, and *PeAP10*-At1g01300. This suggests that these genes likely existed in the genome of the most recent common ancestor shared by grape and *Arabidopsis* (Figure 4). The interpretation of synteny became more complex in instances where segmental duplications in populus aligned with just one *Arabidopsis* gene, or conversely, when a single gene from populus was associated with several *Arabidopsis* genes (Figure 4). The first situation was not detected, but the second included *PeAP7*-AT1G44130/AT1G77480, *PeAP10*-AT1G01300/AT3G61820, *PeAP17*-AT1G62300/AT4G04450, *PeAP29*-AT2G23950/AT4G30520, *PeAP45*-AT5G45130/AT4G19640, *PeAP49*-AT1G02500/AT4G01850 (Figure 4). Within this group, two orthologs of *PeAP17* found in Arabidopsis (At1g62300, At4g04450) were not classified as *AP* genes. Nevertheless, both possess a WRKY domain, unlike *Pe*AP17, which lacks the ASP domain. It is possible that *PeAP17* has experienced several notable chromosomal rearrangements and fusions.

### 2.5. Subcellular Localization Prediction of PeAPs

Since details regarding subcellular locations can offer insights into predicting protein functions, our analysis suggests that all 22, 1, 2, 3, and 10 *Pe*APs are anticipated to be found in the chloroplast, extracellular space, nucleus, plasma membrane, and vacuole, respectively, with a high reliability index (RI > 6). However, exceptions were noted for *Pe*AP2, *Pe*AP7, *Pe*AP11, *Pe*AP14, *Pe*AP18, *Pe*AP25, *Pe*AP29, *Pe*AP31, *Pe*AP32, *Pe*AP35, *Pe*AP44, *Pe*AP47, *Pe*AP53, *Pe*AP54, *Pe*AP55, *Pe*AP57, and *Pe*AP61, which may be situated in the nucleus, plasma membrane, and extracellular regions (Table S4). Notably, *Pe*AP1 was exclusively found in the chloroplast (RI = 14), while the remaining *Pe*APs were predicted to be present in at least two different subcellular organelles (Table S4). The identified *Pe*AP proteins in the *P. euphratica* genome exhibited various subcellular distributions, which showed functional diversity and may be involved in woody plant developmental growth.

### 2.6. Spatial and Temporal Expression of PeAPs in Various Developmental Tissues of P. euphratica Under Salt Treatment

**Figure 5:**
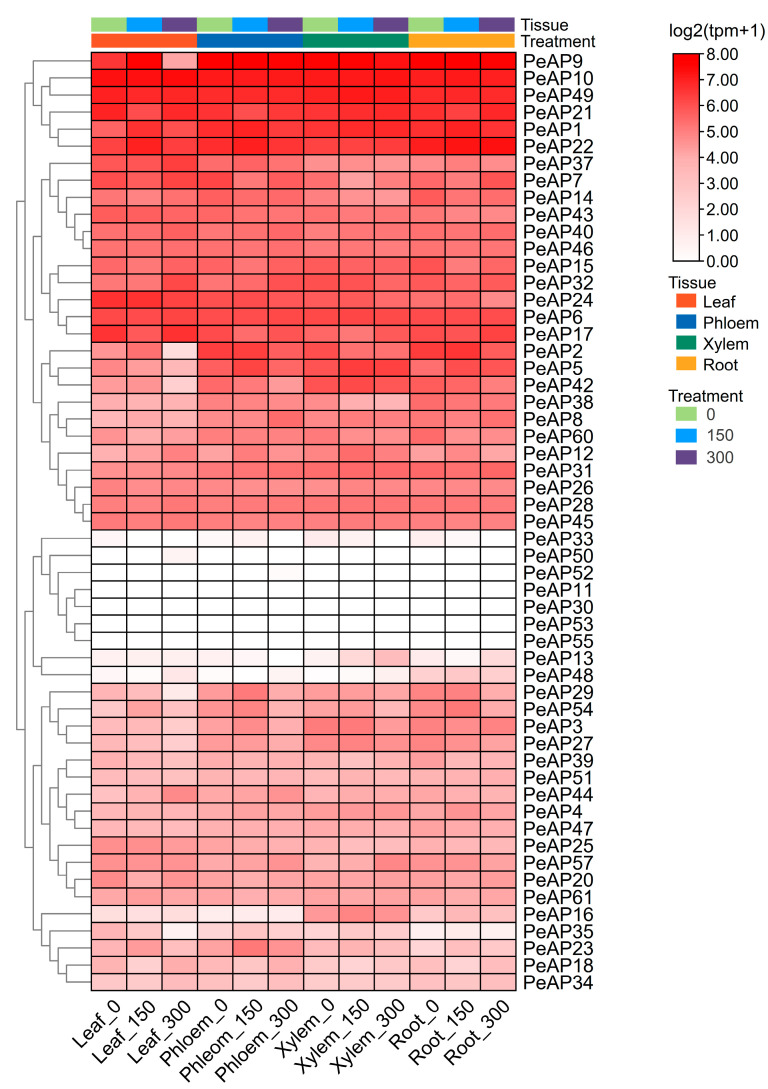
Expression profile of aspartic protease genes in *P. euphratica* under different concentrations of salt stress. Color scale at the right of the dendrogram represents expression values.

To assess the transcriptional levels of *PeAP* genes across various organs, a heatmap utilizing RNA-seq data (Table S5), which were downloaded from the NCBI database (https://www.ncbi.nlm.nih.gov/bioproject/PRJNA390611 (accessed on 1 March 2025)), was created. Our analysis revealed intricate, specific, and overlapping expressions of *PeAPs* in different tissues subjected to salt treatment. The organs were categorized into four distinct types: leaf, phloem, xylem, and root. The findings indicated that 55 *PeAP* genes exhibited unique expression patterns under varying salt stress conditions in these tissues. The transcriptional levels of these genes fluctuated among the different organs. For instance, 8 *PeAPs* (*PeAP11*, *PeAP13*, *PeAP30*, *PeAP33*, *PeAP50*, *PeAP52*, *PeAP53*, *PeAP55*) were found to be minimally detectable in whole plants; conversely, the transcriptional signals of 4 *PeAP* genes (*PeAP9*, *PeAP10*, *PeAP22*, *PeAP49*) were significantly prominent in the overall plantlet. Furthermore, transcript levels showed variability even within the same organs. In the four tissues of *P. euphratica*, the expression levels of the *PeAP* gene family members were generally low (Figure 5). Certain genes were exclusively expressed in specific tissues or organs; for example, *PeAP48* was only detectable in the root. Overall, the *PeAPs* transcriptional levels differed among tissues and played a role in *P. euphratica* development.

### 2.7. Prediction of Cis-Acting Elements in the Promoter Regions of the PeAP Genes

**Figure 6:**
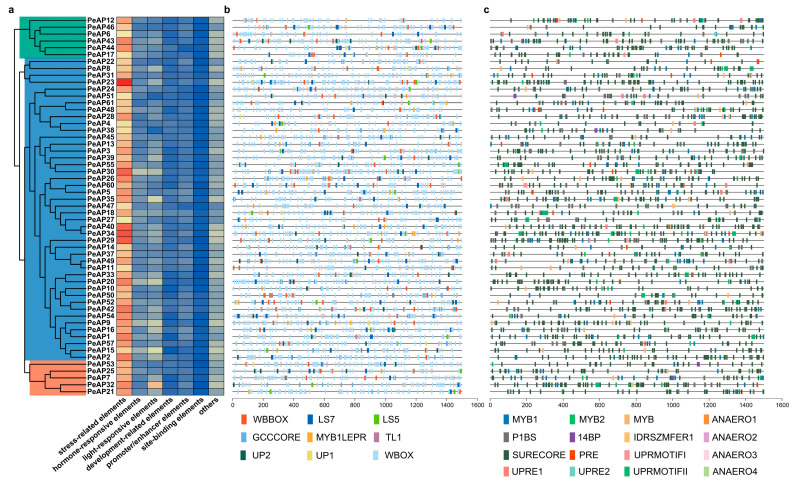
Distribution of cis-elements in aspartic protease gene promoters of *P. euphratica*. (**a**) Number of cis-elements detected in the promoter region of each aspartic protease gene. Elements were grouped into 7 types. (**b**) Distribution of biotic stress related cis-elements in aspartic protease gene promoters of *P. euphratica*. (**c**) Distribution of abiotic stress related cis-elements in aspartic protease gene promoters of *P. euphratica*.

To explore the possible functions of *PeAP*s, a database of plant promoters was utilized to locate the area situated 1.5 kb upstream from the transcription initiation site of *PeAPs*. In total, we discovered 14,488 transcription factor binding sites (TFBS) that fell into 93 distinct categories within the promoter regions of all *PeAPs*. The identified elements encompassed those related to stress, hormone responsiveness, light response, development, as well as promoter and enhancer functions, site-binding characteristics, and various other elements (Figure 6a). In comparison to other elements, there was a notable increase in the prevalence of hormone-responsive and stress-responsive elements (Figure 6a).

*Populus euphratica* exhibits remarkable stress tolerance in natural environments. TFBS analysis further revealed that the distribution abundance of stress-responsive TFBSs is significantly higher than other types of TFBSs (Figure 6a). Therefore, we initially focused on both biotic stress-related TFBSs and abiotic stress-related TFBSs in subsequent analyses. Within the various elements associated with biotic stress, the W-box, which is specifically identified by *WRKY* DNA binding proteins activated by salicylic acid (SA), exhibited a notable enrichment in the *PeAP*s promoter, surpassing other transcription factor binding sites related to biotic stress by over eight times. WB-box *cis*-element, which shows high similarity to W-box, also occurred in almost all *PeAP* promoters except for *PeAP35*. LS7, which acts as the positive salicylic acid-inducible element, is nearly ubiquitous across all *PeAPs*, with the exception of *PeAP12* and *AP31* (Figure 6b). SURECORE, as a sulfur starvation-responsive *cis*-element, exhibits an exceptionally high distribution density in the *PeAP* gene promoter region, significantly surpassing that of other *cis*-elements. PHR-1 binding site (P1BS), which accounts for phosphate starvation responses, occurred across approximately all promoter sequences of *PeAP*s except for *PeAP55*. *MYB1*, which is associated with dehydration-responsive genes, was distributed across all *PeAPs* and occurred more than 7 times in *PeAP7*, *PeAP43*, and *PeAP49* (Figure 6c). Alongside the *cis*-elements mentioned earlier, ABRE elements categorized as hormone-related *cis*-elements were also widely identified within the promoter regions of *PeAPs* genes. These findings indicate that *PeAPs* could influence stress adaptability, responsiveness to phytohormones, and developmental growth.

### 2.8. Regulatory Network Mediated by P. euphratica AP Genes

Studying gene function is crucial for building networks that illustrate interactions among gene families. In this study, we developed a regulatory network governed by the aspartic protease genes of *P. euphratica* using STRING. Out of the 55 *PeAP*s analyzed, 19 *AP*s were found to have significant interactions with other proteins, achieving a high confidence level (combined score > 0.5) (Figure S3). The other 9 interacted items correspond to 9 *P. euphratica* proteins listed in Table S6. Eight genes were detected as orthologues in *Arabidopsis*, except for PeuTF10G02367, without any orthologue in *Arabidopsis* in BLASTP results under a coverage > 60% threshold. Phylogenetic analysis supported that all conserved homologous genes in this cluster encode enzymes, with PeuTF05G00504 and PeuTF07G01029 specifically annotated as peroxidases (Figure S3). Their functional dominance suggests a potential role in mediating drought and osmotic stress tolerance in *P. euphratica*. All interacting proteins are enzymes, suggesting that aspartic protease may functionally cooperate with other enzymes.

## 3. Discussion

The gene family of aspartic proteases is crucial for how plants respond to stress and regulate their development [7]. In our research, we performed an extensive analysis across the genome of the *AP* gene family in *Populus euphratica*, a salt-tolerant woody species known for thriving in extreme saline conditions [25,26]. We identified 55 *PeAP* genes and classified them into three distinct groups based on conserved domain architecture. Our findings suggest that *PeAPs* have undergone significant evolutionary conservation while retaining functional plasticity, which may contribute to their roles in stress adaptation, particularly under saline conditions.

Phylogenetic analysis revealed that *PeAPs* cluster closely with their homologs in *Arabidopsis thaliana* and *Vitis vinifera*, indicating a conserved evolutionary trajectory among dicots [27]. However, *PeAPs* also exhibit lineage-specific expansions, particularly in clades associated with stress-responsive genes. This observation aligns with previous reports showing that gene family expansion in extremophytes like *P. euphratica* often correlates with enhanced abiotic stress tolerance [28,29,30]. Given that aspartic proteases are critical for stress memory in plants, the presence of *PeAPs* may imply a role in long-term salt adaptation.

Promoter analysis of *PeAPs* revealed an abundance of stress- and hormone-responsive *cis*-elements, including *ABRE* (abscisic acid-responsive), *MYB* (drought-inducible), and *G-box* (light-responsive) motifs. This finding suggests that *PeAPs* are tightly regulated by multiple signaling pathways, consistent with their putative roles in stress adaptation [18,26]. Notably, many *PeAPs* contain jasmonic acid (JA)- and salicylic acid (SA)-responsive elements, reinforcing the hypothesis that APs participate in defense responses [31]. Given that JA and SA pathways are crucial for salinity tolerance [32], the hormonal regulation of *Pe*APs may be a key factor in *P. euphratica*’s exceptional salt resilience.

Our expression profiling demonstrated that several *PeAP*s are significantly upregulated under salt stress, particularly AP12 and AP23. These genes share homology with *Arabidopsis AP*s known to mediate osmotic stress responses [33]. Intriguingly, *PeAP11* and *PeAP49* exhibit structural similarity to *CDR1* (Constitutive Disease Resistance 1), an *AP* involved in pathogen defense [34], suggesting a dual role in biotic and abiotic stress responses.

*AP*s are known to degrade pathogenesis-related proteins, which can modulate stress-responsive pathways [35]. In *P. euphratica*, certain *PeAPs* may process pro-proteins involved in ion sequestration or reactive oxygen species (ROS) scavenging. For instance, *PeAP27* contains a conserved domain similar to *AtASP38* (*AtPCS1*) in *A. thaliana*, which regulates programmed cell death under oxidative stress [14]. Given that salinity induces ROS accumulation [36], *PeAP*-mediated protein processing may enhance antioxidant capacity, thereby improving salt tolerance.

Additionally, some *PeAPs* may influence cell wall remodeling, a critical process in salt adaptation. APs have been implicated in the deposition of secondary cell wall [37]. Since *P. euphratica* exhibits unique cell wall modifications under salt stress [38], *PeAPs* could contribute to maintaining structural integrity under osmotic stress. Unlike herbaceous models, woody plants possess a more complex *AP* gene family, likely due to their long-life cycles and perennial growth habits [37,38]. Our comparative analysis with *A. thaliana AP*s revealed that *PeAPs* have undergone fewer tandem duplications but more transposon-mediated expansions, possibly reflecting adaptive evolution in extreme environments. This divergence suggests that *P. euphratica* has evolved specialized *AP* isoforms optimized for saline habitats. The identification of salt-responsive *PeAPs* provides promising candidates for improving salinity tolerance in sensitive crops. Overexpression of *AP12* or *AP23* in salt-sensitive poplar varieties could validate their functional roles. Given that *APs* are involved in multiple stress pathways [39], manipulating their expression may offer a multifaceted approach to enhancing abiotic stress resilience.

This research offers the initial comprehensive examination of the *AP* gene family within *P. euphratica*, emphasizing its possible function in adapting to saline environments. The conservation of stress-responsive *cis*-elements, coupled with the induction of specific *PeAPs* under salinity, underscores their importance in extremophyte biology. Future research should focus on functional characterization using CRISPR/Cas9-mediated knockout or overexpression studies to elucidate the precise mechanisms by which *PeAPs* confer salt tolerance.

## 4. Materials and Methods

### 4.1. Identification of AP Genes in Populus euphratica

A comprehensive analysis was conducted involving the retrieval of 51 *Arabidopsis AP* protein sequences. These sequences served as queries in a targeted search for potential *AP* genes within *Populus euphratica* reference genome [40]. This genome encompasses the chromosomal scale components associated with both male and female *Populus euphratica*, providing critical insights into the molecular mechanisms underlying gender determination and the phenomenon of sexual duality. The identification process was facilitated by the use of BLASTP (v2.15.0) software, which enabled the researchers to effectively pinpoint and analyze the relevant genetic sequences. Then, HMMER (v3.3.1) program and PFAM (PF00026) were employed to identify candidate AP protein in *P. euphratica*. The intersection of genes from both methods was treated as a highly confidential candidate *P. euphratica AP* genes. Protein sequences of all candidate *AP* genes were sent to NCBI-CDD to confirm that they have the complete Asp domain [41]. The molecular weight, hydrophobic characteristics, and isoelectric point were determined using an R package Peptides (v2.4.6).

### 4.2. Multiple Sequence Alignment and Phylogenetic Analysis

A total of six *Populus euphratica* candidate aspartic protease (AP) proteins lacking complete ASP domains were excluded from phylogenetic analysis and subsequent investigations. The remaining 55 *Pe*APs, along with 30 Vitis vinifera APs (*Vv*APs), 51 *Arabidopsis thaliana* APs (*At*APs; pepsin-like type), barley nucellins (GenBank accession no. AAB96882.1), tobacco CND41 (BAA22813.1), cardoon cardosin A (CAB40134), and porcine pepsin A (NP_999038.2), were subjected to multiple sequence alignment using MAFFT. Conserved sites were subsequently extracted from the alignment using Gblocks (v0.91b) [42] to remove divergent and ambiguously aligned regions. Phylogenetic reconstruction was performed with IQ-TREE (v2.2.0) employing the Neighbor-Joining (NJ) method under default parameters. The resulting phylogenetic trees were visualized and annotated using iTOL [43], with color-coding applied according to established aspartic protease classification groups.

### 4.3. Gene and Protein Structure Analysis

Genomic sequences along with their associated coding sequences (CDSs) for *Pe*APs were obtained from the genome of *Populus euphratica* utilizing SeqKit and TBtools. In order to discover conserved motifs within PeAP proteins, we utilized the Multiple Expectation Maximization for Motif Elucidation (MEME) suite (v5.2.0), applying these parameters: Optimal motif width ranging from 10 to 50 amino acids; Maximum number of motifs set to 10; All other configurations were kept at their default settings. For each PeAP protein, functional domains were predicted and annotated using NCBI’s Conserved Domain Database (CDD) and SMART (Simple Modular Architecture Research Tool). Finally, schematic representations of protein structures were generated using TBtools.

### 4.4. Cis-Elements Identification

The promoter sequences of *PeAP* genes, measuring 1.5 kb and located upstream of the TSS site, were extracted by bedtools from the genome sequence according to the position record in genome annotation file. Promoter sequences were submitted to PlantPAN 4.0 web tool for TFBS identification. All identified TFBS were further classified into 6 groups as stress-related, hormone-responsive related, light-responsive, development-related, promoter/enhancer elements, and others according to TFBS functional annotations. All stress-related cis-elements were further validated by manual inspection. The cis-elements distribution visualized by TBtools.

### 4.5. Analysis of Transcriptional Profiles

The sequencing data for the transcriptome of *Populus euphratica* were sourced from the NCBI Sequence Read Archive (SRA) with the accession number SRP116293. The complementary DNA (cDNA) libraries derived from both control and salt-stressed samples of leaf, phloem, xylem, and root tissues have the following accession numbers: SRX3139499, SRX3139976, SRX3139977, and SRX3140050, respectively. Initially, the raw sequencing reads were evaluated for quality using FastQC (v0.11.9) and subsequently processed through fastp (v0.23.2) utilizing default settings for adapter trimming and quality filtering. The STAR aligner (v2.7.10b) was utilized with default parameters to align high-quality reads to the reference genome of *Populus euphratica* (female). Gene expression quantification was performed using feature Counts (v2.0.3) to generate read counts for all annotated genes across each sample.

### 4.6. Analyses of Synteny in PeAPs

Information regarding the physical locations of the *PeAP* genes was obtained from the genome database of *P. euphratica*. To examine the homology of *AP* genes between *P. euphratica* and *A. thaliana*, the MCScanX program was utilized with its default settings. All synteny information about *P. euphratica* and *A. thaliana* was visualized by TBtools.

### 4.7. Prediction of 3-Dimensional Structures and Interaction Network of PeAP Proteins

The three-dimensional (3D) configurations of PeAP proteins were visualized using the SWISS-MODEL database, from which 3D diagrams were obtained. A 3D protein model was constructed with complete confidence for all genes that were positively predicted, while the coverage of residues ranged between 78% and 98%. The 3D structures from each group were randomly selected to make a comparison among *A. thaliana*, *V. vinifera*, and *P. euphratica*. Moreover, we created a protein–protein interaction network of 55 PeAP proteins based on their homologs in *Populus trichocarpa* versus *A. thaliana* using the STRING online database (https://string-db.org, accessed on 20 April 2025).

## 5. Conclusions

This research presents the initial comprehensive examination of the *Aspartic protease* (*AP*) gene family within Populus euphratica, revealing 45 *PeAP* genes that may contribute to salt tolerance. Phylogenetic and expression analyses revealed that several *PeAPs* (e.g., *PeAP22* and *PeAP51*) are strongly induced by salt stress and may function in stress adaptation through protein processing, ROS scavenging, or cell wall remodeling. The findings offer valuable genetic resources for improving salt tolerance in woody crops through molecular breeding. Future work may involve CRISPR-Cas9 knockout validation of *PeAP22* and *PeAP51* to dissect their functional roles in ionic homeostasis or ROS detoxification.

## Data Availability

The genome sequence and annotation files of *Populus trichocarpa* (*female*) were downloaded from the CNCB (PRJCA006811) (Chromosome-scale assemblies of the male and female *Populus euphratica* genomes reveal the molecular basis of sex determination and sexual dimorphism.). The transcriptome sequencing data of *Populus euphratica* were obtained from the NCBI Sequence Read Archive (SRA) under project accession number SRP116293. The accession numbers for the complementary DNA (cDNA) libraries obtained from the controls and the salt-stressed leaf, phloem, xylem and root samples are SRX3139499, SRX3139976, SRX3139977 and SRX3140050, respectively.

## Supplementary Materials

The following supporting information can be downloaded at: https://www.mdpi.com/article/10.3390/plants14131930/s1. Table S1: Detailed information about predicted APs proteins in *P. euphratica*; Table S2: List of APs CDS and protein sequences from *A. thaliana*, *V. vinifera*, and *P. euphratica*; Table S3: The MEME motif sequences and lengths of the *PeAPs*; Table S4: Subcellular localization prediction of *Pe*AP proteins; Table S5: The RNA-seq data of *PeAPs*; Table S6: The information of interacted proteins with *Pe*APs. Figure S1: Three dimensions structure comparison of proteins from *A. thaliana*, *Vitis vinifera*, and *P. euphratica* in each group. Figure S2. Protein sequence alignment of the Populus euphratica aspartic proteinases. The alignment was done using ClustalW and then coloured with Genedoc software using the default parameters. Figure S3. Protein-protein interacting network of *PeAPs* predicted by STRING.
