# Supplementary material for: Genome-Wide Identification and Expression Analysis of Aspartic proteases in Populus euphratica Reveals Candidates Involved in Salt Tolerance
Source: Plants (Basel). 2025 Jun 23;14(13):1930. doi: 10.3390/plants14131930 (PMC12252427; doi:10.3390/plants14131930)
Supplement: Supplementary file 1 [file plants-14-01930-s001.zip › Figure S1.pdf]

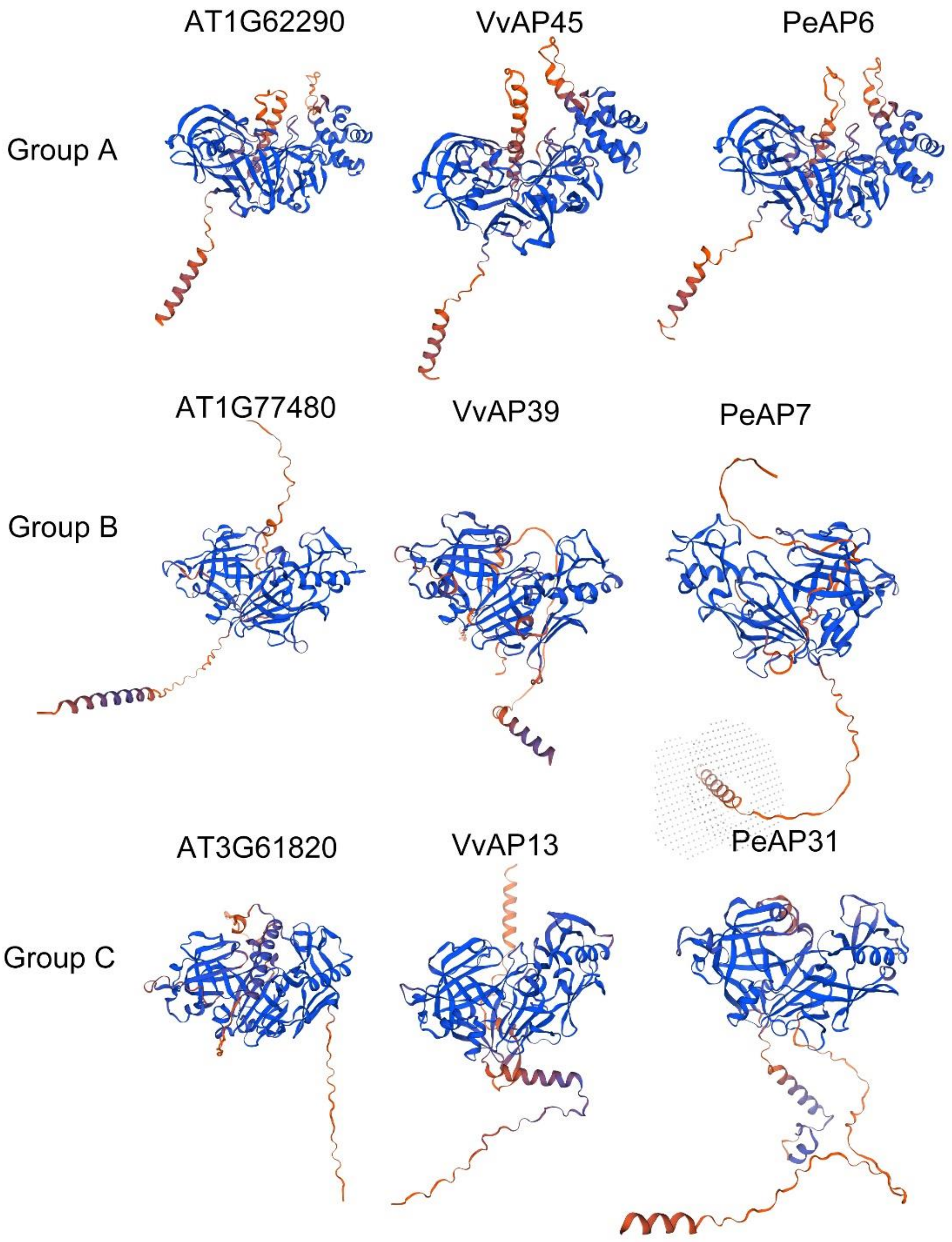

Figure S1 Three dimensions structure comparison of proteins from *A. thaliana*, *Vitis vinifera*, and *P. euphratica* in each group.
