# Supplementary material for: Genome-Wide Identification and Expression Analysis of Aspartic proteases in Populus euphratica Reveals Candidates Involved in Salt Tolerance
Source: Plants (Basel). 2025 Jun 23;14(13):1930. doi: 10.3390/plants14131930 (PMC12252427; doi:10.3390/plants14131930)
Supplement: Supplementary file 1 [file plants-14-01930-s001.zip › Figure S2.pdf]

```
                          *        20         *        40         *        60         *        80         *       100         *       120         *       140         *       160
PeAP1     : -------------MKAYLFSLAFLY----LNTRGQGTTVKVFHVFSPQSPFRPSKPVSWEDSVLQMLAEDQARLHFLSNLAGRKS-----------WVPIASG-RQIVQSPTYIVKANVGTPAQTFLMALDTSNDAAWIPCNGCVG-----CSS--TVF : 123
PeAP2     : ------MVQAKPPFPSKLLLLSLLVSILATRWENGATILEMKHKDSCSGKILD-----WNKKLQKHLIMDDFQLRSLQSRMKSII--SGRNIDDLVDAPIPLTS-GVRLQTLNYIVTVELGGQK--MTVIVDTGSDLSWVQCQPCKR--CYNQQD--PVF : 140
PeAP3     : ---MAPSYSLLLCLSLCFYHILISTS--------QTLFLPLTHSLSKTQFTSAHHLIKSTSTISITRFRHRHQ--KNTHNHRQVS------------LPLSP---------GSDYTLSFTLDSQPISLYLDTGSDLVWFPCQP-FECILCEGKALASTP : 125
PeAP4     : ---------------------------------MG-PLFPAGILIAVVVFHATVVLSSFPATLHLERGVPAS-HKLKLSQLKERDR-RMLQSSGGGVVDFPVQGTFDPFLVGLYYTRLQLGSPPRDFYVQIDTGSDVLWVSCSSCNGCPVSSGLHPLNFF : 124
PeAP5     : ASMTSLCFVLALAMFTIFFSPAFSTSRRA--------------FRVRLKHVD-------------LGKNLTKLERIRHGVKRGRN-------LKTMALVASSSAPVLPGNGEFLMKLAIGTPPETYSAILDTGSDLIWTQCKPCTQ--CFHQST--PIF : 121
PeAP6     : ---MGTILK-PVAAATLFLCFLLLPMISS-LSPPNDGLIRIGLKKRKYERNNRLAAKLE------SKEGDSIKKYHLLRNLGGDAED----------DIVSLKN----YMDAQYFGEIGIGTPPQKFTVIFDTGSSNLWVPSSKCYFSVACYFHSISGFF : 135
PeAP7     : --------------------------------MEKKSKRIVS-LVTMMLLFFIVMAANFRGCFSAASQTP----------------PTTPANDRVGSSVFFRVTGNVYPTGHYSVILNIGNPPKAFDLDIDTGSDLTWVQCDAPCKGCT----KLDKLY : 106
PeAP8     : ----MECDRVVVLVVVVLLCFQFEASIGLLIHRFSDEAKSLSISRKGNASGDLWPKRYSFKYFQLLLGNDLKRQRMKLGSQKNQ-----LLFPSQGSQALFFGN----ELDWLHYTWIDIGTPNVSFLVALDAGSDLLWVPCDCIQCAPLSASYYALSEY : 147
PeAP9     : ------MDLPHCAATFFLFALLFST----CATQSDNSDLSVIPIYSKCSPFVPPKQDSWVNTVITMASKDPERLRYLSTLADQKTT-----------SVPIAPG-QQVLKIANYVVRVKLGTPGQQMFMVLDTSNDAAWVPCSGCTG-----CSS--TTF : 131
PeAP10    : EGKARN------APALLFFSFTLVFLSLSDSTFSEASTTTSLSVQLHHLDAL-SSDKTPQDLFNSRLARDASRVKSLTS-LAAATGR---RARGPGFSSSVISG--LAQGSGEYFTRLGVGTPARYVFMVLDTGSDIVWIQCAPCKK--CYSQTD--PVF : 143
PeAP11    : -----MEAPLSFAISTVLLCVASFA----VYADNAGFTLEPIHRDSSESPFHNPQEIIHLQRVNNALRRSVNRVHHFDPTAAASVSP-----------KAAESE--ITSASGEYLMSLSFGTPPLTVM------------------------------- : 106
PeAP12    : ---MGTQFT-AIWIALLLS----FPVVLS---ARDDGLMRIGLKKKKLDQLGRRVVPGPVNFIPKEEGGGASKPAATKKYYNLGETE----------DIVALKN----YLDAQYYGEIAIGTPPQTFTVIFDTGSSNLWVPSSKCYFSLACYFHSISGFF : 135
PeAP13    : ---MATSHSLLLCFILCFTHVFISTS--------QTLFLPLIHSLSKTQFTSTHHLLKSTSTRSTARFHHHHHHNNKNSHKHRQVS------------LPLSP---------GSDYTLSFTINSQPISLYLDTGSDLVWFPCQP-FECILCEGKALASTP : 127
PeAP14    : ------MATTSFGFVTIVICFISLSPFP-AASPDPGFSLDLIHRDSPLSPLYNPNH-TDFDRLRNAFSRSISRVNVFK-TKAVDIN-------------SFQND--LVPNGGEYFMKMSIGTPLVEVIVIADTGSDLTWVQCLPCDPC--YRQKY--PLF : 132
PeAP15    : ----------------------------------------MKHKDSCSGKILD-----RNKKLQKHLIM---------------------------------------------------------------------------------------- :  24
PeAP16    : QSLNPQSSFSFATMKAYLFSLAFLF----LSTEGLGTTVKVFHVYSSQSPFRPSKPISWEESVLQMMAKDQARLQFLSSLVARKS-----------VVPIASG-RGVIQSPSYIVKAKVGTPPQTLLMALDNSYDAAWIPCKGCVG-----CSS--TVF : 136
PeAP17    : GVNMGVNLQ-AIVG-FVFLSFLLFAVVSS---ASNDGLLRIGLKKVKLDKNNRIAARLDS----KETLRASIRKYNLCSNLG-ESED----------DIVALKN----YLDSQYYGEIGVGSPPQKFTVIFDTGSSNLWVPSSKCYLSVACYFHSISGFF : 136
PeAP18    : ----MASPPLSKETMLLLLLLLLS--------SLFLTTQETQLKNDSLSFSFPLTSLPLSPQTSPSFYSSFISQAKKTPALKSAAS-----------PYNYRS--RFKYSMILLVSLPIGTPPQSQQMILDTGSQLSWIQCHKKVPR--KPPPS--TVF : 131
PeAP20    : ---------MSASRALLLLVVAMLLHLVTIPNDSGSEGKQKLKLVHRDKVSFNKKFHNHSHDFHARMQRDVKRVVSLIRRVSSGSAA---SYGVEDFGSEVVSG--MDQGSGEYFVRIGVGSPPRSQYMVIDSGSDIVWVQCKPCTQ--CYHQTD--PLF : 142
PeAP21    : -------------------------------MGKEKEGFCVVGVLVLFLFLGSSVTSDDRQKRWRKAMVSG-----------------SSMLIN-RVPSSIVLPLHGNVYPNGYYNVTLNIGQPSKPYFLDVDTGSDLTWLQCDAPCVQCL----EPHPYY : 108
PeAP22    : ----MEAPFLLLILVMSSFLVQNTAEL-ALIHRFSKEYKEVSISRGVDVKGTWWPEKKSKEYYQILVSSDLKRQKLKLG-PHYQ-----LLFPSQGSKTMSLGN----DFGWLHYTWIDIGTPHVSFMVALDSGSDLFWVPCDCVQCAPLSASHYDLSEY : 145
PeAP23    : HSFHVRHHKFPFLFLLFLFVLIESCSGFGIHHRFSDPVKGMFGVDG------LLPVKDSVPYFQVMAHRDRVIHGRRLAASAGGDNNKTPLTFYYGNETFRIN-----SLGFLHYANVSVGTPSVSFLVALDTGSNLLWLPCDCSSCVHSLGS--KLNIY : 147
PeAP24    : ----------------------------MARLIQLTILFIQFYYYYYITTTAHGVSSNGTTFLLDSSRSAMILPLFLSPPNLCTK-LLQRSNANAPPNARMRLHDDLLINGYYTTRLWIGTPPQRFALIVDTGSSVTYVPCSSCEQCG----------- : 119
PeAP25    : --------------------------------MEKKSKRSVS-LMMMMFLFFMVISADFQGCFSAASQTP----------------SSTPANDRLGSSVFFRVTGNVYPTGYYSVILNIGNPPKPFDFDIDTGSDLTWVQCDAPCKGCT----KRDKLY : 106
PeAP26    : --MLSRSFIVLVLVLSLLFSGAFEAIAGIDDEGGEEGKEAKQTVKLHLKHRSKDRKSEGKESFVESTNRDLARIQTLHTRIIEKKNQKRPEKQIKTVVATAASPSGVSLGSGEYFMDVFIGTPPKHYSLILDTGSDLNWIQCVPCHD--CFEQNG--PYY : 154
PeAP27    : SIMVSTSILCTSYTNPYLKSLFLLF--------HTVLTLLLIMESQHCLSSSPKMLILPLKTQVIPS----------------GSVP-----------RSPNKL--PFHHNVSLTVSLTVGTPPQNVSMVIDTGSELSWLHCNKTLS------YP--TTF : 115
PeAP28    : --------------------------------MR-GLWPP-FLVIILVSFMFVSAVYCASLLHLERAFPLNNHGLELHQLRARDR-RLLRGFVGGVVDFSVQGSSDPYLVGLYFTKVKLGSPPREFNVQIDTGSDVLWVCCNSCNNCPRTSGLGQLNFF : 124
PeAP29    : ---MDTVFVLVSSLPLIFSTHFALTVANNFSSIQPTRLVTKLIHRDSIVSPYYRSNDTVADRTERTMKASLARLSYLYAKIERDFDI----------DLWLNLHP---SASEPLFLVNFSMGQPPVPQLAIMDSGSSLLWIQCAPCKSCSQQIIGP---MF : 142
PeAP30    : KYSTSMASSRFLFLLSSLILISSSTPTPAATAPTSTITIPLSAPSSTKLIISSKNPWGALNHLASLSLSRAHHIKSPETKFSLLKT-------------PLFPR-----SYGGYSISLNFGTPPQTTKLVMDTGSSLVWFPCTSRYLCSRCDFPNGIPTF : 142
PeAP31    : -----------------------------MHHRFSDSFKNWSGLTR------NWPEKGSFEYYAALAHRDQMLRGRRLSDADAS------LAFSDGNSTFRIS-----SLGFLHYTTVELGTPGVKFMVALDTGSDLFWVPCDCSRCAPTHGASYELSIY : 114
PeAP32    : ------------------------------------------MIG--------KQRWRKAMMSS-----------------SSMLIN-RVPSSIVLPLHGNVYPTGFYNVTLNIGQPSKPYFLDVDTGSDLTWLQCDAPCVQCT----EPHPYY :  82
PeAP33    : ---------------------------------------------------------MHRDVKRVASLIHRLSSGSAA---KYEVENFGSDVVSG--MNQGSGEYFVRIGLGSPPRSQYMVIDSGSDIVWVQCKPCTQ--CYHQTD--PLF :  85
PeAP34    : -----------------MAFLQLL--------VQLYISFILLQSKHCLSSNQPPIVLALRTQKHRTPIS---------TPRLFSTTS-----------KTTDKL--LFHHNVTLTVSLKAGTPLQDITMVLDTGSELSWLHCKKEPN------LN--SIF : 105
PeAP35    : -----------MVSLSLLFHLLLLAFVDL----------------TTEYLKLPLLHKTPFPTPLQSLSSDLQRLSLLHHSHHRHQN-----HRRASSKSPLISG--ASSGSGQYFVSIRLGSPPQTLLLVADTGSDLTWLRCSACKTNCSIHPPG--STF : 124
PeAP37    : --MANIVYLRSPLLVVILFLLFSFHAS--PLAKKDGFGVALIHRDSPNSPLYNADE-TPLERFNNAFQRGISRLNLLN---MKQYN-------------GPDSG--VSSDMGQYLLKFSIGSPPADIYGIVDTGSDLMWTQCQPCEQC--YRQIN--PIY : 133
PeAP38    : --------------------------------MGAAPFPAGILIAAVVFHATMVLSSFPATFHLERGITAN-HKMKLSKLKERDR-RMLQSSGVGVVDFPVQGTFDPFLVGLYYTRLQLGTPPRDFYVQIDTGSDVLWVSCGSCNGCPVNSGLHPLNFF : 125
PeAP39    : ---MVSVSSSLLYCLSLLSLFPIIS---------SSITIPLQHPQTNQIPF--QDQYQKLNHLVTTSLARARHLKHPQTTPATTTTT----------TAPLFSH-----SYGGYSVSLSFGTPPQTLSFIMDTGSDIVWFPCTSHYLCKHCSVSSRIQPF : 131
PeAP40    : --------------------------------MNIVTPAIFQRSVNMGALPSKYSVFILENVLHN-------------------CV------------KANDAI--TIRFAIDSERMQRKGSGIRQN--ASRNALPITYNRC-----------FS--CQI :  80
PeAP42    : -----------------------------S--------ELTVELLSRTSIQK-TTHTDYKSLTLSRLQRDSARVKSLVTRLDLAINSTDSEFKSEDLQSPIISG--TSQGSGEYFSRVGNRK--------------------------------- :  82
PeAP43    : -----------MEAIRFLLISICMGAWLGG--SLSSDGLARVGLKKRNLDLN---------------SIHAARITRPQATSFARVTSN-----------EIVYLKN----YLDTQYYGEIGIGSPPQIFTVVFDTGSSNLWVPSSKCLLSITCYFHSVSGFL : 119
PeAP44    : ---MGVNLK-AIGG-FVLLSYLLFAVVLS---ESND---------------------EALRASIRKYNLLGNLG-ESED----------DIVALKN----YFDAQYYGEIGVGTPSQKFTVIFDTGSSNLWVPSSKCYLSVACYFHSISGFF : 108
PeAP45    : ------MAIMLNNITTFLFFLLVNS-----LLFYSTQSLARPRNPNSLILGLTPASRASHPTHPKASTSSRKKLTDVLDMMEPLR----------------------EVRDGYLISLSIGTPPQVIQVYMDTGSDLTWAPCGNLSFDCIECDDYMMASF : 126
PeAP46    : LLRLNSILHPRMGNKILLKAFCLWALTYF-LPASSNGLVRIGLKKRHLDLQTIKDARIAR-----QEGKAGVGASSRVHDLG--SSD----------DIIPLKN----YLDAQYLGEIGIGSPPQNFTVIFDTGSSDLTWVPSSKCYFSGTS---VVK--- : 132
PeAP47    : --------------------------------MAMPKLEVPLLFLFFFFFFLAPTPSRAETFGPPLVSPIQK----------------------------------DASTLQYIITAYLQSPKETKLLLDLGASYIWINCDDYNSSSYRHIPC--TLL :  90
PeAP48    : ------------------------------MRMMMAEALSCVLLLTMMISFGIVSANNG--VFSVKYKYAGL--QRSLSDLKAHDD-RILAG-----VDLPLGGIGRPDILGLYYAKIGIGTPTKDYYVQVDTGN---------------------------- :  95
PeAP49    : --MAAFQSPLSLALAIALLCVSSFG----IYARKVGFTVDLIHRDSPLSPFYNSEE-TDLQRINNALRRSISRVRHFDPIAAASVSP-----------KAAESD--VTSNGGEYLMSLSLGTPPFKIMGIADTGSDLIWTQCKPCERC--YKQVD--PLF : 136
PeAP50    : ------------------------------------------------------------------------------------------------------------------------------------------------------------ :   -
PeAP51    : -----------------------------MANSSSSSSLVIS---YSLILLNLYAIVS--STSDFNNRHHPMILPLLLSTPNISAHRHLQNS---ELPNARMRLFDDLLSNGYYTTRLFIGTPPQEFALIVDTGSTVTYVPCSSCEQCG----------- : 112
PeAP52    : SEPASKPFLLFCALIFLFFTSTLPRDLPDTPSFSNSSLSFSLELHPRETIYK-IHHKDYKSLVLSRLHRDTARFNSLTARLQLALEDT--EIKPEDLSTPVTSG--TSQGSGEYFTRVGVGNPARQFYMVLDTGSDINWLQCQPCTD--CYQQID--PIF : 151
PeAP53    : --MESDDDQSPQFKGVVIISLPPPDNPSLSSRLFLGTPRKLLSFLFISLFALAIYSSLFTNTFQELKSNNNDDDDDQKPKSFVFPLYASSNAAAAMDSSTIFPVRGNLYPDGLYFTYMLVGSPPQPYYLDFDTGSDLTWIQCDAPCTSCA----KANAWY : 154
PeAP54    : --MESISCKSLEAMMGLLWIMVCYLSLASQSINQSSIHLNVYHVHGHGSSLTP----NSSSLLSDVLLHDEEHVKALSDRLANKG--SGHLLEPNS-ASIPLDP-GLSIGSGNYYVKLGLGSPPKYYAMVLDTGSSLSWLQCQPCVVY-CHAQAD--RLY : 147
PeAP55    : -----MASSLLLFFLSFLILITSSKSTPT------TITIPLSAPSFTELIVSSKNPWGSLNHLASLSLSRAHHIKSPKTNFSRIKT-------------PLFPR-----SYGGYSISLSFGTPPQTTKFVMDTGSSLVWLQCTSRYFCSECNFPN-VPTF : 130
PeAP57    : TPISSVSLTMKCFLYAYFLCLCLLFSLEKLSNNDNKASLKVVHKHGPCSKLSLN-EASAAPTHTEILLQDQSRVKSIHSRLSNS---GKDVKVTDS-TTIPAKD-GSIVGSGNYIVTVGLGTPKKDLSLIFDTGSDITWTQCQPCARS-CYKQKE--QIF : 151
PeAP60    : ANMSSLSFVVALAIFALVFSHAFSTSRRV-------------FRVKLKHVD-------------SGKNLTKFERIQHGVKRGRH-------FKAMALVASSNAPVLPGNGEFLMKLAIGTPPATYSAIMDTGSDLIWTQCKPCTQ--CFDQPT--PIF : 121
PeAP61    : --------------------------------MDLRRLVALLMVLLVAELGLVSFSLGNVVFKVQHKFAGR--IRNLTEFKAHDA-RLLSS-----VDLPLGGNGHPAETGLYFAKIGLGNPSKDYYVQVDTGSDILWVNCIGCDKCPTKSDLGKLTLY : 119
cardosin  : ---MGTSIK-ANVL-ALFLFYLLSPTVFS---VSDDGLIRIGLKKRKVDRIDQLRGRRA------LMEGNARKDFGFRGTVR--DSG----------AVVALTN----DRDTSYFGEIGIGTPPQKFTVIFDTGSSVLWVPSSKCINSKACRAHSITGFF : 130
nucellin  : --------------------------------------------------------MAAMWSP-----------------LLLLLPLGPSSAIKFPLEGNVYPVGHFYATLNIGEPAKPYFLDVDTGSNLTWLECHHPVHGCKGCHPRPHPYY :  80
CND41     : HSLMSTGSYFLLFSSSAFLLILLSFSVEKKRRG---ASLEVVNRQGPCTLLNQ--KGAKAPTLTEILAHDQARVDSIQARITDQSYDNKKKSVKDSKANLPAQS-GLPLGTGNYIVNVGLGTPKKDLSLIFDTGSDLTWTQCQPCVKS-CYAQQQ--PIF : 151
                                                                                                                                                g p        d gs     c

                          *       180         *       200         *       220         *       240         *       260         *       280         *       300         *       320
PeAP1     : NSVTSTTFKSLGCDAPQCKQVP--------NPICG--GSTCTWNTTYGG---STILSNLTRDTIALS-TDIVP--------GYTFGCIQKTTG------SSVPPQGLLGLGRGPLSFLSQTQGLY---KSTFSYCLP--SFRTLNFS-GTLRLGP----- : 244
PeAP2     : NPSTSPSYRTVLCSSPTCQSLQSATGN---LGVCGSNPPTCNYVVNYGD--GSYTRGELGTEHLDLGNSTAVS--------NFIFGCGRNNQG------LFGGASGLVGFGRSSLSLISQTSAMF---GGVFSYCLP--RAETEAS--GSLVMGGN---S : 271
PeAP3     : PPKLSKTATPVSCKSSACSAAHSNLPSLESIETSDCQKHSCPQFYYAYG--DGSLIARLYRDSISLPLSNPTN----LIVNNFTFGCAHTALA---------EPIGVAGFGRGVLSLPAQLATLPPHLGNQFSYCLVSHSFDSDRLRRPSPLILGRY-DH : 269
PeAP4     : DPGSSPTASLISCSDQRCSLGL-----QSSDSICAAQNNQCGYTFQYGD--GSGTSGYYVSDLLHFDTILGGSV-MNNSSAPIVFGCSTLQTGD-LTKPD-RAVDGIFGFGQQDMSVISQLASQGLTPR-VFSHCLKGDDSGGGILVLGEIVEP------ : 267
PeAP5     : DPKKSSSFSKLSCSSQLCEALPQS--------SCSNG---CEYLYSYGD--YSSTQGILASETLTFGKAS---------VPNVAFGCGANNEGS-----GFSQGAGLVGLGRGPLSLVSQLK------EPKFSYCLT--SAD-DAKTSTLLMGSLA--SV : 243
PeAP6     : SQDHVKVGDLVVKNQEFIEATREPSVT---ILGLGFQEISVGKAVPVWY--NMVDQGLVKEPVFSFWFNRNVDE---KEGGEIVFGGVDPDHYKGEHTYVPVTQKGYWQFDMGDVLIGGQTSGFCASGCAAIADSGTSLLAGPTTIITEVNHAIGATGVV : 287
PeAP7     : K--PKNN--RVPCASSLCQAIS-----TGQNYNCDIPTEQCDYEVQYAD--LGSSLGVLLSDYFPLRLNN-----GSLLQPRIAFG-CGYDQKY-RGPHSPPDTAGILGLGRGKASILSQLRTLGITQN-VVGHCFSR--VTGGFLFFGDHHFPPS---- : 241
PeAP8     : SPSLSSTSRHLSCDHQLCEWG----------SNCKNPKDPCPYIFNYDDFENTTSAGILVEDKLHLASVGDHTA-RKMLQASVVLGCGRKQGGS---FFDGAAPDGVMGLGPGDISVPSLLAKAG-LIQNCFSLCFDE--NDSGRILFGDRGHASQ---- : 286
PeAP9     : LPNASTTIGSLDCSGAQCSQVRGFS-----CPATG--SSACLFNQSYGG--DSSLTATLVQDAITLA-NDVIA--------GFTFGCINAVSG------GSIPPQGLLGLGRGPISLISQAGAMY---SGVFSYCLP--SFKSYYFS-GSLKLGP----- : 256
PeAP10    : NPTKSRSFANIPCGSPLCRRLDSP--------GCSTKKQICLYQVSYGD--GSFTYGEFSTETLTFR-GTRVG--------RVALGCGHDNEG------LFIGAAGLLGLGRGRLSFPSQIGRRF---SRKFSYCLV--DRSASSKPSYMVFGDSA--IS : 271
PeAP11    : ------------------------------------------------------------------------------------------------------------------------------------------------------------ :   -
PeAP12    : SQDSVEVGDLVVKNQDFIEATKEPGVT---ILGLGFQEISVGNAVPVWY--NMVNQGLVKEKVFSFWLNRNVEG---EEGGEIVFGGVDPNHYKGEHTYVPVTHKGYWQFDMGDLLIGTETTGLCAGGCKAIADSGTSLLAGPTTVITQINNAIGASGIV : 287
PeAP13    : PPKLSKTATPVSCKSSACSAVHSNLPSLESIEISDCRKHSCPQFYYAYG--DGSLIARLYRDSIRLPLSNQTN----LIFNNFTFGCAHTTLA---------EPIGVAGFGRGVLSLPAQLATLSPQLGNQFSYCLVSHSFDSDGVRRPSPLILGRY-DH : 271
PeAP14    : DPSRSSSYRHMLCGSRFCNALDVSE------QACAMDTNICEYHYSYGD--KSYTNGNLATEKFTIGSTSS-RP---VHLSPIVFGCGTGNGGT-----FDELGSGIVGLGGGALSLVSQLSSIIK--G-KFSYCLVP--LSEQSNVTSKIKFGTD---- : 266
PeAP15    : --------------------------------GVCGSNPPTCNYVVT----------------MVMGPTQAV----------------RKNQG------LFGGASGLVGLGRSNLSLISQNSPMF---GGVFSYCLP--TTEAEAS--GSLVMGGN---S : 104
PeAP16    : NTLKSTTFKTLGCRAPQCKQVT--------NPICG--GSTCTWNTTYGS---STILSNLTQDRIALS-MDPVP--------NYAFGCIQKATG------SSVPPQGLLGLGRGPLSFLSQTQNLY---KSTFSYCLP--SFRTLNFS-GSLRLGP----- : 257
PeAP17    : SNDAIEVGGLVVKDQEFIEATKEPGIT---ILGLGFKEISVGDAVPVWD--NMIKHGLIKEPVFSFWLNRNAED---EEGGEIVFGGMDPKHYKGKHTFVPVTRKGYWQFNMGDVHIGDKPTGYCASGCAAIADSGTSLLAGPATIITMINQAIGASGVV : 288
PeAP18    : DPSLSSSFSVLPCNHPLCKPRIPDFT----LPTSCDLNRLCHYSYFYAD--GTLAEGNLVREKITFSTSQSTPP---LILG---------CAED-------ASDDKGILGMNLGRLSFASQAKIT------KFSYCVPTRQVRPGITPTGSFYLGEN---- : 257
PeAP20    : DPADSASFMGVSCSSAVCD--QVE--------NAGCNSGRCRYEVSYGD--GSSTKGTLALETLTLG-RTVVQ--------NVAIGCGHMNQG------MFVGAAGLLGLGGGSMSFVGQLSRER---GNAFSYCLV--SRVTNS-NGFLEFGSEA--MP : 267
PeAP21    : R--PSNN--LVACTDPICQSLQ-----SNGDHRCENP-GQCDYEVAYAD--GGSSFGVLVRDTFNLNFTS-----EKRHSPLLALGLCGYDQFP-GGSHHPID--GVLGLGKGKSSIVSQLSSLGLVRN-VIGHCLSG--HGGGFLFFGDDLYDPS---- : 241
PeAP22    : SPSQSSTSKQLSCGHRLCDMG----------QNCKNPNQSCPYRINYYT-ESTSSSGLLVEDIIHLASGGDDTL-NTSVKAPVIIGCGMKQSGG---YLDGVAPDGLLGLGLQEISVPSFLAKAG-LIQNSFSMCFNE--DDSGRIFFGDQGPTTQ---- : 283
PeAP23    : SPNTSSTSEKVPCNSTLCSQTQ--------RNRCPSDQSDCPYQVVYLS-NGTSSTGYFVQDLLHLIS--DDSQ-SKAVDAKITFGCGKVQTGT---FLTGGAPNGLFGLGMSNISVPSTLAHNG-YTSGSFSMCFSP--DGIGRISFGDKGSTGQ---- : 285
PeAP24    : ---RHQDPKFQPDLSSTYQSVK-----CNIDCNCDDENQQCVYERQYAE--MSTSSGVLGEDIISFGNLS------ALAPQRAVFGCENMETGD-LYSQH---ADGIMGLGRGDLSIVDHLVDKGVIND-SFSLCYGGMGIGGGAMVLGGISPPS---- : 253
PeAP25    : K--PKNN--LVPCSNSICQAVS-----TGENYHCDAPDDQCDYEIEYAD--LGSSIGVLLSDSFPLRLTN-----GTLLQPKMAFG-CGYDQKH-LGPHPPPDTAGIMGLGRGKASILSQLRTLGITQN-VVGHCFSR--ARGGFLFFGDHLFPSS---- : 241
PeAP26    : DPKESSSFRNIGCHDPRCHLVSSPDP----PLPCKAENQTCPYFYWYGD--SSNTTGDFALETFTVNLTSPTGKSEFKRVENVMFGCGHWNRG------LFHGASGLLGLGRGPLSFSSQLQSLY---GHSFSYCLV--DRNSDTNVSSKLIFGED--KD : 295
PeAP27    : DPTRSTSYQTIPCSSPTCTNRTQDFP----IPASCDSNNLCHATLSYAD--ASSSEGNLASDVFHIG-SSDISG---LVFGCMDSVFSSNSDE-------DSKSTGLMGMNRGSLSFVSQLGFP------KFSYCIS------GTDFSGLLLLGES---- : 242
PeAP28    : DSTSSSTAGRVHCSDPICTSAV-----QTTATQCSSQTDQCSYTFQYGD--GSGTSGYYVSDTLYFDAILGQSL-IANSSALIVFGCSAYQSGD-LTKTD-KAVDGIFGFGQGELSVISQLSTRGITPR-VFSHCLKGEGNGGGILVLGEILEP------ : 267
PeAP29    : DPSISTTYDSLSCKNIICRYAPS---------GECDSSGQCVYNQTYVEG--LPSVGVIATEQLIFGSSDEGRN----AVNNVLFGCSHRNGNY-----KDRRFTGVFGLGSGITSVVNQMGS--------KFSYCIGNIADPDYSYNQLVLSKGVN---- : 271
PeAP30    : IPKLSSSSNLIGCKNHKCSWLYG----QECDPTTQNCTQSCPPYVIQYG--LGSTAGLLLSETLDFPDK--------KTIPGFLVGCSLFSIR---------QPEGIAGFGRSPESLPSQLG------LKKFSYCLVSHALD-DTPA-SSDLVLDTG-SG : 270
PeAP31    : NPRESSTSKKVTCNNDMCAQ----------RNRCLGTFSSCPYIVSYVS-AQTSTSGILVKDVLHLTT--EDGG-REFVEAYVTFGCGQVQSGS---FLDIAAPNGLFGLGMEKISVPSVLSREG-LIADSFSMCFGH--DGIGRISFGDKGSPDQ---- : 250
PeAP32    : K--PSND--LVAWKDPICQSLH-----TNGDQKHENP-EQCDYEVEYAD--GGSSLGVLVKDAFNLNFTS-----EKRQSPLLALG-CGYDQLP-GGTYHPID--GVLGLGRGKPSIVSQLSGLGLVRN-VIGHCLSG--RGGGFLFFGDDLYDSS---- : 214
PeAP33    : DPADSASFMGVSCSSAVCD--RVE--------NAGCNSGRCRYEVSYGD--GSYTKGTLALETLTFG-RTVVR--------NVAIGCGHSNRG------MFVGAAGLLGLGGGSMSFMGQLSGQT---GNAFSYCLV--SRGTST-NGFLEFGSEA--MP : 210
PeAP34    : NPLASKTYTKIPCSSPTCETRTRDLP----LPVSCDPAKLCHFIISYAD--ASSVEGNLASETFRVG-SVTGPA---TVFGCMDSGFSSNSEE-------DAKTTGLMGMNRGSLSFVNQMGFR------KFSYCIS------DRDSSGVLLLGEA---- : 232
PeAP35    : LARHSTTFSPAHCFSSLCQLVPHPNPN---PCNHTRLHSTCRYEYVYSD--GSKTSGFFSKETTTLNTSSGRE----MKLKNIAFGCGFHVSGPSLIRSSFNGASGVMGLGRGPISFASQLGRRF---GRSFSYCLM--DYTLSPPPTSYLMIGDV--VS : 268
PeAP37    : NPKSSSTYTDISCQSNECHLLDT----------ATCSAESLCNYTYAYAS--SSFTKGVLAKEAITFSSSSGGQP---VSVPDIVFGCGHSNSG------FNEREMGLVGLGGGRPLSLASQIGSYLG--NRKFSYCLVP--FHTDPSISSKMYFGDG---- : 265
PeAP38    : DPGSSPTASLISCSDQRCSLGL-----QSSDSVCSAQNNLCGYTFQYGD--GSGTSGYYVSDLLHFDTVLGGSV-MNNSSAPIVFGCSALQAGD-LTKSD-RAVDGIFGFGQQDMSVVSQLASQGISPR-AFSHCLKGDDSGGGILVLGEIVEP------ : 268
PeAP39    : IPKESSSSKLLGCKNPKCSWIHR----DQDCSIKSCLNQTCPPYLIFYG--SGTTGGVALSETLHLHS---------LSKPNFLVGCSVISSH---------QPAGIAGFGRGLSSLPSQLG------LGKFSYCLLSHRFDDDTKK-SSSLVLDME-QL : 259
PeAP40    : N--KTTKKFRVNDNNGRRKTRTSDLT----LPVTCDPSKLCHFIISYAD--ASSVEGHLAFETFRFG-SLTPPA---TVFG-------SNTEE-------DAKTTGLMGMNRGSLSFVNQMGLR------KFSYCIS------GLDSTGFLLLGEA---- : 198
PeAP42    : -TTKSSLFN---TRHWQCR--SLD--------VSECRNDTCLYEVSYGD--GSYTVGDFVTETITLG-SAPVD--------NVAVGCGHTNEG------LFVGAAGLLGLGGGSLSFPSQIN------ATSFSYCLV--DRDSES-ASTLEFNST---LP : 199
PeAP43    : SQDHVKVGDDIIIDQEFAEVTREGLLA---ILGLAFQDIAVGKATPVWY--NMAEQGHVSQKVFSLWLNRNPSS---ELGGEIVFGGLDWRHFKGDHTYVPVTGGGYWQIQVGDIFIANNSTGLCAGGCAAIVDSGTSFLSGPTRIVAQINHAIGARGIV : 271
PeAP44    : SIDAVEVGNLVVKDQEFIEATKEPSIT---ILGLGFKEIAVGGAVPVWYVYNMIKQGLIKEPLFSFWLNRNVDD---EEGGEIVFGGMDPNHYKGKHTYVPVTQKGYWQFDMGDVIVGDKSTGYCAGGCAAIADSGTSLLAGPTAIITMINHAIGASGVV : 262
PeAP45    : SPSHSSSSHRDSCTSPFCIDVHSSDNPLSTLVKAKCSWPCPPFAYTYGAG--GVVTGTLTRDTLRVHGRNLGVT---QEIPRFCFGCVGSTYR---------EPIGIAGFGRGALSLPSQLGFLR----KGFSHCFLAFKYANNPNISSPLIIGDIA--- : 265
PeAP46    : --TTVQVGDLVVKDQVFVEATKEGSLS---ILGLGFQEISVENVVPCRY--NMMQQDLVDDEVFSFWLNRNPEA---KDGGELVFGGVDPKHFKGKHTYVPVTQKGYWQINMGDFLIGKHSTGLCEGGCAAIVDSGTSLLAGPTPIITEINHAIGAEGLV : 282
PeAP47    : DEFVGCFACLMNCRDSSPNCGDS----------VCVLKPENPFQPSFSGNEQYAPALVDYFSMLTLDNTGSVGGPSSSSPFTFIFSCGYKENLKG----LARGVTGSAGLGRSSISIPVQASAIFHY-PRYFALCLSGSKTRPGAAFVGTKGPYKFG--- : 232
PeAP48    : ------TGKLVPCDQEFCYEIN-----GGQLPGCTTNMS-CPYLEIYGD--GSSTAGYFVKDVVQYARVSGDLK-TTAANGSVIFGCGARQSGD-LGSSNEEALDGILGFGKSNSSMISQLAVTGKVKK-IFAHCLDG-TNGGGIFVIGHVVQP------ : 231
PeAP49    : DPKSSKTYRDFSCDARQCSLLDQ----------STCSSNICRYQYSYGD--RSYTMGNVASDTITLDSTTG-SP---VSFPKTVIGCGHENDGT-----FSNKGSGIVGLGAGPLSLISQMGSSVG--G-KFSYCLVP--LSSLAGNSSKLNFGRN---- : 266
PeAP50    : ------------MQTPLS---------------CSTKKQICLYQVSYGD--GSFTVGEFSTETLTFR-GTRVG--------RVVLGCGHDNEG------LFVGAAGLLGLGRGRLSFPSQIGRRF---NSKFSYCLV--DRSASSRPSSIVFGDSA--IS : 109
PeAP51    : ---KHQDPRFQPDLSSTYRSVK-----CNPSCNCDDEGKQCTYERRYAE--MSSSSGVIAEDVVSFGNES------ELKPQRAVFGCENVETGD-LYSQR---ADGIMGLGRGRLSVVDQLVDKGVIGD-SFSLCYGGMDVGGGAMVLGQISHPP----- : 246
PeAP52    : DPTASSTYAPVTCQSQQCS--SLE--------MSSCRSGQCLYQVNYGD--GSYTFGDFATESVSFGNSGSVR--------NVALGCGHDNEG------LFVGAAGLLGLGGGPLSLTNQLK------ATSFSYCLV--NRDSAG-SSTLDFNSVQ--LG : 274
PeAP53    : K--PRRGD-IVPPKDLLCMEVQ-----RNQKAGYCETCDQCDYEIEYAD--HSSSMGILATDKLLLMVAN-----GSLTQLNFIFG-CAYDQQG-LLLKTLVKTDGILGLSRAKVSLPSQLASQGIINN-VIGHCLTTDVGGGGYMFLGDDFVPRW---- : 292
PeAP54    : DPSVSKTYKKLSCASVECSRLKAATLN---DPLCETDSSACVYTATYGD--TSFSIGYLSQDLLTLTSSQTLP--------QFTYGCGQDNQG------LFGRAAGIIGLARDKLSMLAQLSTKY---GRAFSYCLP--TANSGSSGGGFLSIGS----I : 279
PeAP55    : FPKLSSSSKLIGCKNRRCSMFFG----QECDSTAQNCTQTCPPYIIQYG--SGSTAGLLLSETLDFPDK--------KTIPDFLVGCSIFSIK---------QPEGIAGFGRSPESLPSQLG------LKKFSYCLVSHAFD-ETPT-SSDLVLDTG-SG : 258
PeAP57    : DPSQSTSYTNISCSSSICNSLTSATGN---TPGCA--SSTCVYGIQYGD--SSFSVGFFGTEKLTLTSTDEFN--------NFYFGCGQNNQG------LFGGSAGLLGLGRDKLSLVSQTAQKY---NKIFSYCLP--SSSSST---GFLTFGG----S : 278
PeAP60    : DPKKSSSFSKLSCSSKLCEALPQS--------TCSDG---CEYLYGYGD--YSSTQGILASETLTFGKVS---------VPKVAFGCGEDNEGS-----GFSQGSGLVGLGRGPLSLVSQLK------EPKFSYCLT--SVD-DTKASTLLMGSLA--SV : 243
PeAP61    : DPASSVSATRVSCDDDFCTSTY-----NGLLPDCKKELP-CQYNVVYGD--GSSTAGYFVRDAVQFERVTGNLQ-TALSNGTITFGCGAQQSGG-LGTSG-EALDGILGFGQANSSMISQLAAAGKVKR-AFAHCLDN-VNGGGIFAIGELVSP------ : 260
cardosin  : SQDSVTIGDLVVKEQDFIEATDEADNV---ILGLSFQTISV----PVWY--NMLNQGLVKERRFSFWLNRNVDE---EEGGELVFGGLDPNHFRGDHTYVPVTYQYYWQFGIGDVLIGDKSTGFCAPGCQAFADSGTSLLSGPTAIVTQINHAIGANGVM : 278
nucellin  : T--PADGNLKVVCGSPLCVAVRRDV--PGIPECSRNDPHRCHYEIQYVT---GKSEGDLATDIISVNGRD-----KKR----IAFG-CGYKQEE-PADSPPSPVDGILGLGMGKAGFAAQLKGHKMIKENVIGHCLSS--KGKGVLYVGDFNPPTR---- : 216
CND41     : DPSTSKTYSNISCTSAACSSLKSATGN---SPGCS--SSNCVYGIQYGD--SSFTIGFFAKDKLTLTQNDVFD--------GFMFGCGQNNKG------LFGKTAGLIGLGRDPLSIVQQTAQKF---GKYFSYCLP--TSRGSN---GHLTFGNGNGVK : 282
                                                  c                    g                             g                  g  g         s                       c
```

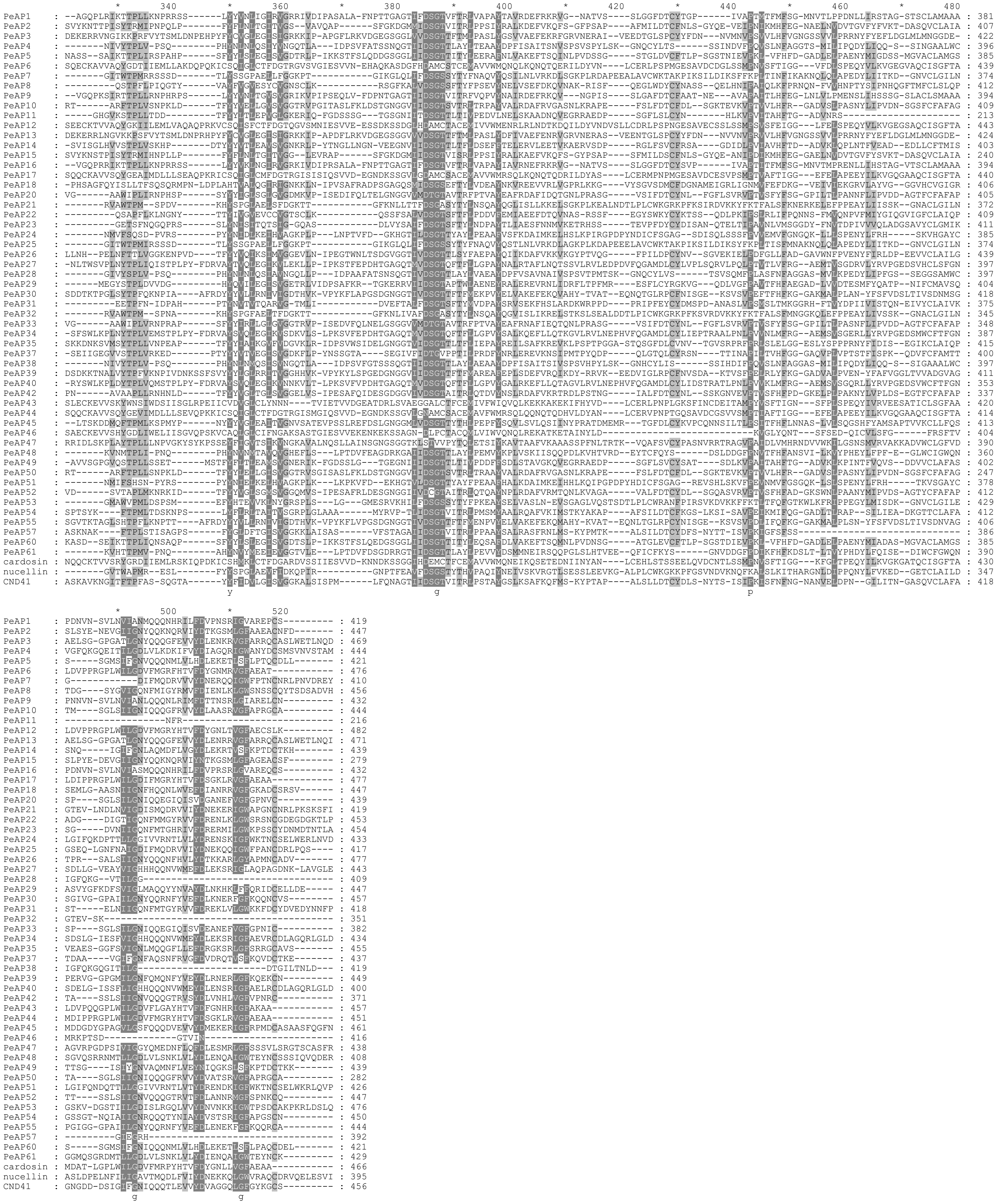

Figure S2 Protein sequence alignment of the *Populus euphratica* aspartic proteinases.
The alignment was done using ClustalW and then coloured with Genedoc software using the default parameters.
