# Supplementary figures and images for: Genome-Wide Identification and Expression Analysis of Aspartic proteases in Populus euphratica Reveals Candidates Involved in Salt Tolerance
Source: Plants (Basel). 2025 Jun 23;14(13):1930. doi: 10.3390/plants14131930 (PMC12252427; doi:10.3390/plants14131930)

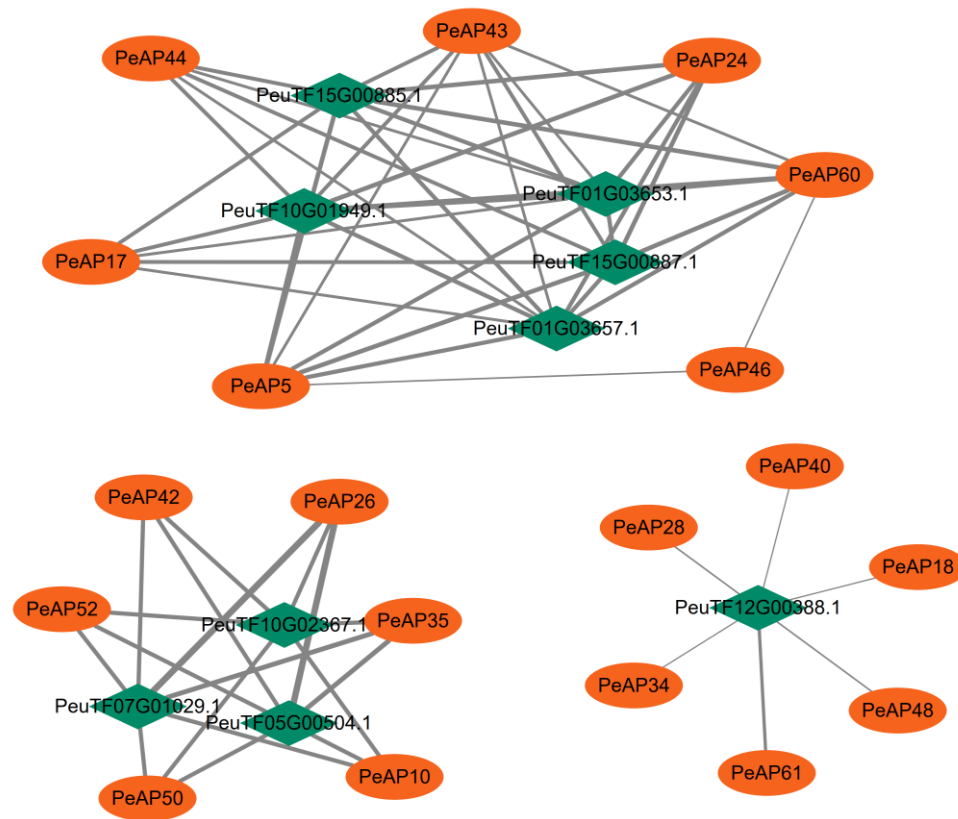

Figure S3. Protein-protein interacting network of PeAPs predicted by STRING.

Supplement: Supplementary file 1 [file plants-14-01930-s001.zip › Figure S3.pdf]
